# Determining the management of pain in people with spinal cord injury by physiotherapists in South Africa

**DOI:** 10.4102/sajp.v78i1.1767

**Published:** 2022-07-27

**Authors:** Bernice James, Mokgadi K. Mashola, Diphale J. Mothabeng

**Affiliations:** 1 Department of Physiotherapy, Faculty of Health Sciences, University of Pretoria, Pretoria, South Africa; 2 Department of Physiotherapy, Faculty of Health Sciences, University of the Witwatersrand, Johannesburg, South Africa

**Keywords:** nociceptive pain, neuropathic pain, pain management, physiotherapy, South Africa, spinal cord injury

## Abstract

**Background:**

Pain after spinal cord injury (SCI) is common, and physiotherapy plays a pivotal role in alleviating pain for people with SCI.

**Objective:**

To determine the modalities that physiotherapists in South Africa use to treat SCI-related pain and the factors that guide the selection of treatment modalities.

**Method:**

A quantitative, cross-sectional design using a self-developed online survey was distributed to physiotherapists belonging to the South African Society of Physiotherapy and the Physiotherapy Association of South Africa. Data were analysed using SPSS v26, where descriptive data were analysed using frequency, percentages, means and standard deviations, and Fisher’s exact tests for inferential analyses. Open-ended questions underwent thematic analysis.

**Results:**

Forty-six responses were received. The most-used modalities were transcutaneous electrical nerve stimulation (29.8%), exercises (27.7%) and joint mobilisations (29.8%). Most physiotherapists used standardised measurements to objectively assess pain characteristics, with the visual analog scale being the most used (70.2%). Except for the cost of treatment, the factors that guided the selection of the modalities included the pain type, onset, duration, location and intensity, pain interference, duration of treatment, patient’s preferences, other treatments that the patient was receiving for pain and psychosocial factors (87.2%).

**Conclusions:**

Local physiotherapists use pain management modalities that are supported by the evidence to treat SCI-related pain.

**Clinical implications:**

This study highlights the common modalities used by physiotherapists to treat SCI-related pain, as well as the selection criteria for the modalities. Owing to the low response rate, we caution against generalising these findings across the SCI pain management field.

## Introduction

A spinal cord injury (SCI) is a neurological condition that results in motor and/or sensory deficits, paralysis and risk of secondary health conditions (SHCs) (Hagen & Rekand 2015). These SHCs are detrimental to functioning and include pain, urinary tract infections, severe muscle spasms, decubitus ulcers and respiratory complications (Mashola & Mothabeng 2019). Pain is a common SHC after SCI and is experienced by 60% – 80% of people with SCI (PWSCI) (Tibbett et al. 2020). In high-income countries, 33% of this pain is often reported as severe pain (Müller et al. 2017), with up to 66% in South Africa (Mashola & Mothabeng 2019). Pain after SCI often commences within the first 6 months after the SCI and is frequently persistent, with the possibility of it being aggravated over time (Ataoğlu et al. 2013; Widerström-Noga et al. 2016). The experience and perception of pain may be intense and can be reported as severe to extreme and interfering with mobility, functioning, activities of daily living (ADLs) and overall independence (Moon et al. 2013), social participation with friends and in the community (Piatt et al. 2016), cognitive function, emotional distress and depression (Ataoğlu et al. 2013) and financial problems (Müller et al. 2017).

Nociceptive pain and neuropathic pain are the main types of pain that can occur in PWSCI (Hussain Khan, Majedi & Asaad Hassan 2019). Nociceptive pain arises because of the activation of nociceptors, as a result of damage to the non-neural tissues, whilst neuropathic pain occurs as a direct result of a disease or lesion affecting the somatosensory system (IASP 2019) such as an SCI. Musculoskeletal shoulder pain is the most common nociceptive pain reported by PWSCI (Cratsenberg et al. 2015), whereas neuropathic pain is more common in the lower limbs below the level of injury (Nakipoglu-Yuzer, Atçı & Ozgirgin 2013; Varghese et al. 2020).

The multifactorial pattern of pain ultimately affects how patients react to pain and respond to pain management. The management of pain in PWSCI is therefore challenging because of the different underlying pain mechanisms and can be further complicated by a variety of emotional, behavioural and social factors that can negatively affect the experience of pain, as well as the individual’s response to pain (Guy et al. 2016). For example, the severity of pain is influenced by various factors such as genetics, comorbidities, current psychological state, prior experience of pain and socio-economic circumstances (Stanos et al. 2016). Treatment is rarely aimed at all the associated factors of pain. Despite the challenges, management of pain is essential and treatment interventions include task modifications, therapeutic and psychological treatments and pharmacological and surgical options.

Therapeutic interventions such as physiotherapy are recommended as first-line treatment, with a wide range of therapeutic interventions such as thermotherapy, electrotherapy, massage, exercises, pain education and advice on self-management (Van Straaten et al. 2017; Widerström-Noga et al. 2016). Physiotherapy treatments are planned based on a detailed evaluation of the patient with regards to the pathway of pain, peripheral and central conditions and any underlying psychological factors. Mild-to-moderate physical activity (such as stretching and strengthening exercises) has been shown to have positive effects on nociceptive pain after SCI (Franz et al. 2019). The benefits of exercises are that they can be adapted for each individual, can be performed independently and are likely to be associated with minimal side effects. Furthermore, exercise is associated with decreased depression and fewer symptoms of anxiety (Geneen et al. 2017; Polaski et al. 2019). Exercise to manage pain needs to be appropriately prescribed and performed correctly to prevent pain flare-ups and injury. There are various exercise-based interventions that are targeted to relieve pain after SCI. They range from aquatic exercises (hydrotherapy), aerobic exercises and resistance (strengthening) exercises (Geneen et al. 2017) to result in exercise-induced hypoalgesia. The type of exercise and the duration, intensity and frequency of the exercises are important in exercise prescription and play a role in whether pain relief is achieved or not (Polaski et al. 2019). Exercise, when performed correctly, reduces pain severity and improves physical function, although it may need to be prescribed in conjunction with other pain management techniques for complete pain relief, such as stretches, medication or transcutaneous electrical nerve stimulation (TENS) (Geneen et al. 2017; Hussain Khan et al. 2019; Polaski et al. 2019).

Although electrotherapy is known to reduce pain in able-bodied individuals (Boldt et al. 2014; Fuentes et al. 2010), some heat-based electrotherapy techniques such as ultrasound and interferential therapy may be contraindicated in PWSCI with pain in noninnervated areas. Transcutaneous electrical nerve stimulation is the most widely used electrotherapy modality, and is a simple, noninvasive treatment extensively used by physiotherapists to treat pain (Bi et al. 2015; Norrbrink Budh & Lundeberg 2004). Conventional TENS uses low intensity (≤ 10 Hz) and high frequency (up to 50 Hz or ≥ 100 Hz), which is one of the most-used TENS parameters, as it is capable of selectively exciting the low-threshold, non-noxious afferent nerve fibres (Aβ fibres) to inhibit the pain-related dermatomes (Gibson, Wand & O’Connell 2017; Mokhtari et al. 2020). The TENS mechanism is explained by the pain gate theory, where the gate can be closed by the activity of the large-diameter Aβ fibres, thus preventing the transmission of the second-order nociceptive noxious information. The closed gate then results in low noxious information reaching the brain from the spinal cord, thus decreasing the pain experienced (Bi et al. 2015; Mokhtari et al. 2020). Transcutaneous electrical nerve stimulation is applied on the skin to activate nerve fibres and induce the release of endogenous opioids. Together with the modification of electrical transmission and blood vessel dilation, neuropathic pain relief is achieved, thus making TENS an effective modality to treat SCI neuropathic pain, with side effects occurring very rarely (Celik et al. 2013; Gibson et al. 2017; Hussain Khan et al. 2019; Mokhtari et al. 2020).

International studies have investigated nonpharmacological pain management for PWSCI (Boldt et al. 2014; Norrbrink Budh & Lundeberg 2004). However, there is limited local evidence for the physiotherapy management of SCI-related pain. A literature search of MEDLINE (PubMed), conducted on 20 May 2022, yielded very few South African studies, and no relevant study investigated the interventions used by physiotherapists to manage SCI-related pain (Online Appendix 1, Table 1-A1). The objective of our pilot study was therefore to determine the interventions that local physiotherapists use for the management of pain in PWSCI and the factors that guide physiotherapists in choosing their modality.

## Methods

A quantitative, descriptive, cross-sectional approach was used. An online capture sheet was created and loaded on Qualtrics^®^ as an online survey to record the participants’ demographic information, as well as the modalities used to treat pain in PWSCI (Online Appendix 1, Online survey). The questions about the treatment selection criteria were guided by the international SCI basic pain data set (Widerström-Noga et al. 2016). The targeted study population was physiotherapists treating SCI-related pain after SCI, from both the South African public and private sectors. A nonprobability convenience sampling method was used, considering the accessibility of the participants, time and cost factors. The Health Professions Council of South Africa reported a total of 7734 registered physiotherapists in 2018 (HPCSA 2018). Spinal cord injury rehabilitation is a small component within the physiotherapy field, with an estimated 10% of all physiotherapists practising in SCI (i.e. 773 physiotherapists). However, due to the data collection being online, this study aimed to achieve a response rate of 56% (*n* = 432) of the sampled physiotherapists, as guided by Baruch (1999). The online survey consisted of 19 questions and included close-ended questions about demographic details and the factors that guided the choice of modality. Open-ended questions were included for participants to report their modality of choice for treating pain in SCI and their suggestions for the treatment of acute and chronic nociceptive pain and acute and chronic neuropathic pain (Online Appendix 1, Online survey).

Qualtrics^®^ was used to create an online version of the capture sheet, which was sent to physiotherapists via the South African Society of Physiotherapists (SASP) and the Physiotherapy Association of South Africa (PASA). The SASP and PASA are professional membership bodies for physiotherapists in South Africa. A pilot study was conducted for content and face validity, as well as to check the practicality of the survey. The pilot study sample size was determined as 10% of the total sample size. Therefore, the online survey was sent to eight physiotherapists who fit the inclusion criteria. The pilot study was used to determine whether the respondents understood the questions and to ensure that the survey met the ethical requirements for the study, such as the anonymity of the participants (Thabane et al. 2010). The pilot study also verified that the web link to the online survey was working and enabled the authors to observe how the respondents answered the open-ended questions. Responses from the pilot study were included in the main study analysis, as there were no changes made. The survey was made available to the participants from January to May 2020. A biweekly reminder was sent to the participants, and the survey was closed by May 2020. Participants consented to participate in the study by submitting the survey responses.

Physiotherapists, including community service physiotherapists, from South Africa, who were involved in SCI rehabilitation, were included. We excluded participants who, although they opened the survey link, did not complete and submit the survey.

Data were analysed using SPSS version 26 and involved a descriptive component presenting the frequencies and percentages, together with Fisher’s exact test to determine the association between demographic profile, choice of treatment and selection criteria. The level of significance was set at *p* < 0.05. Open-ended questions were analysed using inductive thematic content analysis.

### Ethical considerations

Our study received ethical approval from the Faculty of Health Sciences Research Ethics Committee of the University of Pretoria (reference number: 785/2019).

## Results

### Demographic results

A total of 113 physiotherapists opened the capture sheet. However, only 46 of them completed and submitted the capture sheet. The 57 incomplete responses were excluded and we used the 46 complete responses. The consenting physiotherapists had a mean age of 36.9 years (SD 10.49), with most of them between the age of 20 and 40 years (*n* = 30, 65.2%). Most of the physiotherapists were female (*n* = 34, 73.9%), working in a clinical setting (*n* = 38, 82.6%) and in the private sector (*n* = 25, 54.3%). Most physiotherapists had undergraduate degrees (*n* = 34, 73.9%) with, on average, 13.23 years of experience (SD 10.50). Some of the physiotherapists worked in private specialised rehabilitation hospitals (*n* = 13, 28.3%). Many physiotherapists had less than 10 years of experience in the SCI field (*n* = 35, 76%), had treated fewer than 10 PWSCI (*n* = 43, 93.5%) and did not have a special interest in SCI pain management (*n* = 17, 37%) (Table 1).

**TABLE 1:** The frequency and percentage of the physiotherapist’s demographic profile.

Description	*n*	%
**Age**		
20–30	16	34.8
31–40	14	30.4
41–50	9	19.6
51–60	7	15.2
**Gender**		
Male	12	26.1
Female	34	73.9
**Job description**		
Clinician	38	82.6
Academic	6	13.0
Other	2	4.3
**Province**		
Eastern Cape	1	2.2
Free State	3	6.5
Gauteng	33	71.7
KwaZulu -Natal	1	2.2
Limpopo	3	6.5
Mpumalanga	2	4.3
North West	0	0
Northern Cape	0	0
Western Cape	3	6.5
**Area of employment**		
Public sector	20	43.5
Public tertiary/academic hospital	12	26.1
Public specialised rehabilitation hospital	2	4.3
Public Secondary/District hospital	3	6.5
Public Primary hospital /clinic	3	6.5
Private sector	25	54.4
Private hospital	7	15.2
Private specialized rehabilitation hospital	13	28.3
Private practice	5	10.9
University	1	2.1
**Highest qualification**		
Bachelor’s degree	34	73.9
Master’s degree	11	23.9
Doctoral degree	1	2.1
**Years of experience**		
0–10 years	24	52.2
11–20 years	10	21.7
21–30 years	7	15.2
31–40 years	5	10.9
**Experience in SCI**		
0–10 years	34	73.9
11–20 years	7	15.2
21–30 years	2	4.3
31–40 years	3	6.5
**Number of SCI patients treated in a month**		
0–10	43	93.5
11–20	2	4.3
21–30	0	0
31–40	1	2.2
**Special interest in SCI pain management**		
Yes	17	37.0
No	29	63.0

### Modalities used to treat pain in people with spinal cord injury

TENS was the modality used most to treat pain (*n* = 14, 29.8%), followed by exercises (*n* = 13, 27.7%) and joint mobilisations (*n* = 10, 21.3%).

#### Most-used modality: Transcutaneous electrical nerve stimulation

The main factors that guided the participants in selecting TENS were type of pain (*n* = 41, 87.2 %); psychosocial factors (cultural considerations, depression and lifestyle factors [*n* = 41, 87.2%]) and intensity of pain (*n* = 40, 85.1%), as shown in Table 2. There was a significant association between postgraduate qualifications and TENS (Fisher’s exact = 30.416, *p* = 0.043). A significant proportion who cited TENS as their most-used modality held a bachelor’s degree (71.4%). We also found a significant association between a participant’s special interest in pain in PWSCI and using TENS to treat the pain (Fisher’s exact = 20.486, *p* = 0.043).

**TABLE 2:** Factors that guided the selection of modalities (*N* = 46).

Description	TENS				Exercises				Joint mobilisations			
	Yes		No		Yes		No		Yes		No	
	*n*	%	*n*	%	*n*	%	*n*	%	*n*	%	*n*	%
Type of pain (neuropathic or nociceptive)	41	87.2	2	4.3	41	87.2	2	4.3	41	87.2	2	4.3
Onset of pain (sudden or gradual)	34	72.3	9	19.1	38	80.9	5	10.6	37	78.7	6	12.8
Duration of pain (acute or chronic)	38	80.9	5	10.6	41	87.2	2	4.3	37	78.7	6	12.8
Location of pain (above or below the level of injury)	39	83.0	4	8.5	38	80.9	4	8.5	41	87.2	2	4.3
Intensity of pain (mild or severe)	40	85.1	3	6.4	41	87.2	2	4.3	39	83.0	4	8.5
Pain interfering with daily activity	39	83.0	4	8.5	41	87.2	2	4.3	40	85.1	3	6.4
Pain interfering with overall mood	33	70.2	10	21.3	34	72.3	9	19.1	36	76.6	7	14.9
Pain interfering with sleep	31	66.0	12	25.5	35	74.5	8	17.0	36	76.6	7	14.9
Cost of treatment modality	15	31.9	28	59.6	15	31.9	28	59.6	16	34.0	27	57.4
Duration of treatment modality	21	44.7	22	46.8	25	53.2	18	38.3	24	51.1	19	40.4
Patients’ preference (subjective)	33	70.2	10	21.3	32	68.1	11	23.4	31	66.0	12	25.5
Other treatments that patients receive, including past medical history	39	83.0	4	8.5	38	80.9	5	10.6	42	89.4	1	2.1
Psychosocial factors (e.g. cultural considerations, depression, lifestyle factors)	41	87.2	2	4.3	39	83.0	3	6.4	39	83.0	4	8.5

TENS, transcutaneous electrical nerve stimulation.

Fifty-seven per cent of the participants who reported having a special interest in pain in PWSCI used TENS to treat pain.

#### Second most-used modality: Exercises

The main factors that guided the use of exercises as the second most-used modality were the type of pain (*n* = 41, 87.2%), the duration of the pain (*n* = 41, 87.2%), the intensity of the pain (*n* = 41, 87.2%) and pain interfering with daily activity (*n* = 41, 87.2%), as shown in Table 2.

A significant association was found between the age of the participants and exercises (Fisher’s exact = 26.28, *p* = 0.049). Most of the younger participants between the ages of 20 and 30 used exercises as their second most-used modality to treat pain in PWSCI (84.6%).

#### Third most-used modality: Joint mobilisation

The third most-used modality was joint mobilisations (*n* = 14, 29.8%). Participants used the following as the main selection criteria: other treatments the PWSCI is receiving (*n* = 42, 89.4%), type of pain (*n* = 41, 87.2%) and location of pain (*n* = 41, 87.2%) (Table 2). We did not find any association between the participants’ demographic profiles and selecting joint mobilisations to treat pain after SCI.

#### The least-used modalities

Thermotherapy (*n* = 5, 10.9%), acupuncture (*n* = 3, 6.4%), dry needling, kinaesiology taping and cognitive-behavioural therapy (CBT) (*n* = 2, 4.2%, respectively) were the least-used modalities.

### Use of outcome measurements

Most of the physiotherapists used an outcome to measure the pain in PWSCI (*n* = 42, 91.5%) and the visual analog scale was used most to measure the outcomes (*n* = 33, 70.2%).

### Treatment suggestions

**FIGURE 1:**
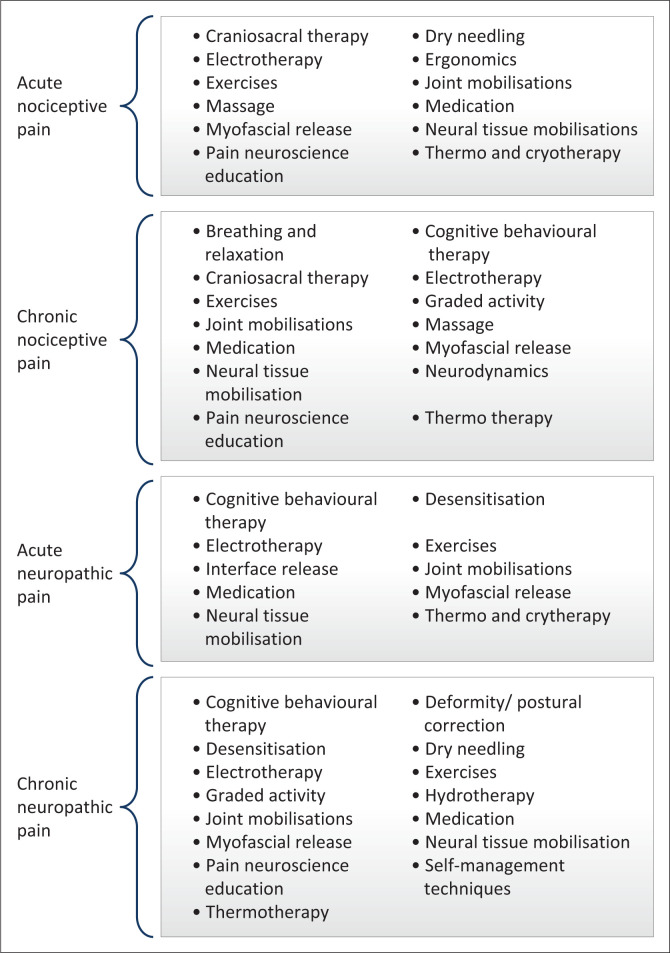
Suggested treatments for the different types of pain.

The participants were asked to suggest treatments for the management of acute and chronic nociceptive pain and acute and chronic neuropathic pain. Thematic analysis identified the treatments, which are shown in Figure 1, with further descriptions included in Online Appendix 1 (Treatments suggested per treatment type by the physiotherapists).

## Discussion

Our study was based on a sample of physiotherapists involved in the management of pain in PWSCI in South Africa. We found that most physiotherapists who participated were young and female. Physiotherapy remains a female-dominated profession (Louw et al. 2021), and younger individuals (less than 45 years old) and women tend to participate in online surveys more than their older or male counterparts (Fan & Yan 2010; Saleh & Bista 2017). Most of the physiotherapists had less than 10 years of experience in the SCI field and treated fewer than 10 PWSCI per month. This demographic picture suggests that our findings may indicate general pain management interventions that physiotherapists use when they have patients with SCI not necessarily those specifically tailored for PWSCI.

Transcutaneous electrical nerve stimulation was the most commonly used modality and it is widely used around the world for providing analgesia, as it is noninvasive, inexpensive and can be self-administered (Dissanayaka, Banerjee & Johnson 2014). It can be used for both the nociceptive and neuropathic types of pain (Grover, McKernan & Close 2018) and was found to be effective in reducing neuropathic pain following SCI (Krumme & Weinmann 2020). The physiotherapists either used TENS as a monotherapy or in combination with joint mobilisations or exercises. Transcutaneous electrical nerve stimulation has been shown to be effective for pain relief when used in combination with other therapeutic modalities such as exercises and thermal treatments (Maeda et al. 2017). The TENS device and the accessories are inexpensive and can be easily bought over the counter or online (Gourav & Mark 2013).

Physiotherapeutic interventions and treatments are planned based on a detailed evaluation of the patient with regards to the pathway of pain, peripheral and central conditions and any underlying psychological factors. Mild-to-moderate physical activity (such as stretching and strengthening exercises) has been shown to have a positive effect on nociceptive pain after SCI (Franz et al. 2019). Physiotherapy exercises, massage and TENS have been shown to reduce pain in PWSCI when used as an adjunct to pharmacological treatment and with fewer side effects (Celik et al. 2013; Gibson et al. 2017; Hussain Khan et al. 2019; Mokhtari et al. 2020).

Exercises were the second most-used modality of the participants. The physiotherapists selected strengthening exercises and flexibility exercises to treat both neuropathic and nociceptive types of pain in PWSCI, and use of these interventions has been reported in the literature to relieve nociceptive and neuropathic pain (Norrbrink et al. 2012; Polaski et al. 2019). Exercise-induced hypoalgesia is characterised by a reduced sensitivity to a painful stimulus, and the effect of exercises on neuropathic pain seen in their study was reported to be comparable to the effects of the antidepressants and anticonvulsant drugs used to treat pain in PWSCI (Norrbrink et al. 2012; Polaski et al. 2019). In addition to these benefits, the minimal cost involved in prescribing exercise therapy makes it popular amongst physiotherapists (Seth 2014). Physiotherapists across South Africa report inefficiencies in the procurement of therapy devices, meagre budgets, a lack of transport to collect and deliver therapy devices and a lack of spare parts and repair technicians (Sherry 2014). In such a scenario, where access to a rehabilitation facility is limited and resources in terms of infrastructure and healthcare professionals are meagre, minimal investment therapeutic modalities such as exercises are invaluable.

Joint mobilisations were the third most-used modality. Adequate assessment is necessary before treatment using joint mobilisations, and the assessments and treatments can be modified to suit each patient (Ali, Sethi & Noohu 2019; Gautam et al. 2014). Joint mobilisations are a type of manual therapy that includes Maitland’s techniques, which involve applying passive and accessory movements to joints, and Mulligan’s approach, which is based on correcting joint malalignments (Ali et al. 2019). A combination of joint mobilisations with exercise provides better analgesia for musculoskeletal pain (Peters et al. 2020). Gross et al. (2015) also agree that mobilisations are most effective for the management of pain in PWSCI when used in combination with other modalities, such as exercises. Although physiotherapists did not specify joint mobilisation in combination with exercise as their third-most used modality, the physiotherapists may use TENS, exercise and joint mobilisation in combination during their sessions, as we asked them to report their first, second and third most-used modalities to treat pain in PWSCI. The use of a combination of therapies is very common in physiotherapy practice, as different treatment approaches have distinct effects (Moseley 2002).

Thermotherapy, acupuncture, dry needling, kinaesiology taping and CBT were amongst the least-used modalities for treating pain in PWSCI. Although thermotherapy has its benefits in reducing pain, it gives the best results when used in combination with exercises or joint mobilisations (El-Tallawy et al. 2021). However, the risk of burns in PWSCI may also have contributed to it being rarely used. Acupuncture and dry needling still lack standardisation and guidelines concerning the regulation of acupoints (El-Tallawy et al. 2021). In addition, acupuncture and dry needling do not form part of the undergraduate physiotherapy curriculum in South Africa, and one needs to undergo a basic course to practise acupuncture and dry needling. Similarly, with regards to kinaesiology taping, contrary to the global trend, kinaesiology taping was amongst the least-used modalities for physiotherapists in South Africa. The absence of reliable evidence regarding the use of kinaesiology taping to alleviate pain in PWSCI might have been an important reason why it is used rarely (Krajczy et al. 2020). Cognitive-behavioural therapy was also amongst the least-used modalities.

### Factors that guided the selection of treatment modalities

#### Transcutaneous electrical nerve stimulation

The physiotherapists who used TENS as their most-used modality chose it based on the type of pain, the duration of pain, the location of pain, the intensity of pain, other treatments that the PWSCI had received and psychosocial factors. Considering these factors when deciding on a treatment strategy is imperative. The type of pain, whether it is nociceptive or neuropathic, is very important in deciding on the treatment modality, and TENS is effective in managing both neuropathic and nociceptive pain (El-Tallawy et al. 2021). Furthermore, TENS is more effective for alleviating pain at the level of injury than for radiating pain (Mark 2014). Contraindications associated with the application of TENS must be monitored against the other treatments that the patient is receiving (Mark 2014).

#### Exercises

Physiotherapists who chose exercises as their treatment modality did so based on all factors except the cost of the treatment modality. The selection of a treatment strategy is based on a proper evaluation of the condition of the patient. History-taking is indeed the first, basic step in the planning of a treatment strategy and is very important to focus on the health problems faced by the patient. This may be concerning the type of pain, the duration of the pain (acute or chronic), impairments, activity limitations, sleep restrictions, restrictions in social participation and personal and environmental factors (Oostendorp et al. 2017). For instance, psychosocial factors are an important indicator of prolonged disability, as they contribute to the transition of an acute condition to a chronic, disabling condition (Malfliet et al. 2019). In such a scenario, the physiotherapist’s awareness of the patient’s psychosocial status can help in the design of a treatment strategy that can alleviate the risks posed by psychosocial factors. Strategies such as group exercise therapy sessions can be arranged. Exercises as part of a wellness programme have been shown to benefit pain and mood in PWSCI (Crane, Hoffman Reyes 2017). According to previous studies, a multimodal approach, including therapeutic interventions, patient education, psychosocial support, an active lifestyle and peer and family support, can reduce the long-term psychological and socio-economic burden of pain in PWSCI (Malfliet et al. 2019). These benefits can be accessed through exercises.

#### Joint mobilisations

Joint mobilisations were selected by physiotherapists based on all factors except the cost of the treatment modality. Factors such as interference of pain with the daily activities of life, interference of pain with sleep, and pain interfering with overall mood are as important as the onset and duration, location and intensity of pain. Good sleep is important for good health and well-being (Gulia & Kumar 2020). It has been established that quality of life is compromised in PWSCI because of pain (Hearn et al. 2015). In line with the recommendations in the literature, the physiotherapists considered the interference of pain with sleep when selecting the treatment modalities. Poor sleep can act as a barrier to an effective pain management programme (Malfliet et al. 2019). Similarly, an overall good mood and a positive attitude can improve the general condition of PWSCI. Pain following SCI has a complex relationship with the mood of the patient (Kennedy & Hasson 2017). Considering how the pain interferes with the daily activities of life is therefore important in planning a treatment strategy. The physiotherapists also considered the mood factor, which is in line with the existing literature.

#### Summary of the factors that guided the treatment selection

Although the aforementioned criteria were considered by physiotherapists, we found that the majority of the physiotherapists did not consider the cost of treatment. Van Rensburg (2014) found that 44% of health expenditure occurs in the private sector, which only serves about 16% of the South African population. For other South Africans, health care is either inaccessible because of geographic location or financial constraints (Morris et al. 2019). The costs of treatments in the private sector are much higher when compared to the rates in the public sector. In a middle-income country like South Africa, where the inequalities between the upper and lower strata of people are broad, most of the population cannot afford health insurance (Mji, Lieketseng & Cloete 2017). Darien et al. (2020) reported that a cost discussion with the patient could cause the patient to withdraw from the treatment plan, display nonadherence to the treatment protocol and miss appointments. The treatment choices of the physiotherapists in our study are reported in the available literature as effective for relieving pain. However, patients who are unable to afford the treatment may miss appointments to avoid debt. Therefore, in the same way that the physiotherapists considered the patient’s preference when selecting the treatment modality, assessing the financial burden of the treatment should also be taken into consideration. For example, although exercises may be considered low cost (Seth 2014), depending on the type of medical aid the patient has, they would be charged approximately R119.20 for the exercises and R178.45 for the first or follow-up consultation fee (e-MD Technologies 2022). Should TENS and mobilisations be included in the session, there would be an additional R119.20 for TENS and R178.45 for joint mobilisations (e-MD Technologies 2022). For PWSCI who have exhausted their out-of-hospital benefits or do not have medical aid, paying approximately R595.30 per session to manage their pain may not be affordable. Most local PWSCI are unemployed and dependent on the government disability grant (Mashola, Korkie & Mothabeng 2022) and may visit their nearest government clinic or hospital if they cannot afford private health care. However, a shortage of equipment or resources has been cited as hampering the management of SHCs in PWSCI; in these cases, they simply try to live with the pain (Pilusa, Myezwa & Potterton 2021a, 2021b).

### Treatments suggested by the physiotherapists in this study

The physiotherapists in this study recommended a variety of treatments to manage both acute and chronic nociceptive and neuropathic types of pain. The efficacy of many of the treatments is supported in the literature, such as exercise (Polaski et al. 2019), pain neuroscience education (Javdaneh et al. 2021), TENS (Bi et al. 2015; Celik et al. 2013; Gibson et al. 2017) and neural tissue mobilisation (Su & Lim 2016). We agree with Hagen and Rekand (2015) that the management of pain should be based on clinical findings so that the correct type of pain is diagnosed (Hagen & Rekand 2015). The onus lies with the physiotherapist to perform an in-depth assessment of not only the pain presentation but also the SCI characteristics. For example, heat therapy to treat neuropathic pain below the level of injury would be contraindicated in PWSCI with complete injuries due to their sensory loss in that area. Active and strengthening exercises would not be possible in noninnervated areas due to motor loss, and the risk of pain medication misuse would need to be determined before advocating for pain medication (Clark, Cao & Krause. 2017).

## Strengths and limitations

To the best of the authors’ knowledge, our study is the first to investigate pain management interventions by physiotherapists in South Africa and adds to the available literature on SCI pain management. Our study had a low response rate, with only 10% of the anticipated sample participating. We recruited participants from the two physiotherapy associations in the country but not the pain management or SCI associations such as PainSA or the Southern African Spinal Cord Association (SASCA) and SCI workgroups. Most of the physiotherapists in this study did not have a special interest in managing pain after SCI, and those who participated may have been providing generic pain management, not specific to PWSCI. Since our study was performed on an online platform, there is a chance that we missed physiotherapists not well versed in technology and only received responses from physiotherapists interested in the subject of our study.

## Recommendations

Our study has identified some interventions that physiotherapists use to manage pain in PWSCI, and these findings may be useful not only in the SCI health field but also to PWSCI who experience pain. Owing to the low response rate, we caution against generalising these results to all physiotherapists in South Africa.

Similar studies with physiotherapists are recommended where the interventions are specified, for example, the parameters of the TENS application; the frequency and intensity of the types of exercises; and the type, grade and duration of the joint mobilisation techniques.

## Conclusion

As in the global management of SCI-related pain, we found that physiotherapists mostly use TENS, exercise and joint mobilisations to treat pain in PWSCI. Except for the cost of treatment, physiotherapists select their treatment modalities based on the pain presentation, interference, duration of treatment, patient’s preference, psychosocial factors and other treatments that the patient receives.
